# Comments on: Safety and efficacy of aminophylline in the prevention of bradyarrhythmia during coronary atherectomy

**DOI:** 10.1016/j.ahjo.2024.100437

**Published:** 2024-08-03

**Authors:** Robert F. Riley, Timothy D. Henry

**Affiliations:** a Overlake Medical Center & Clinics, Bellevue, WA, United States of America; b The Carl and Edyth Lindner Center for Research and Education, The Christ Hospital, Cincinnati, OH, United States of America

Treatment of calcified coronary lesions pose higher risks for procedural failure and peri-procedural complications compared to non-calcified lesions [1,2]. Techniques for optimizing outcomes and procedural safety have evolved over that time and multiple devices are now available for treating these types of lesions, including atherectomy [2,3]. One of the limitations encountered during atherectomy is significant bradycardia and heart block, which is believed to be due to the release of adenosine versus distal embolization and/or subsequent microvascular dysfunction. This was demonstrated and reported during the development of the AngioJet rheolytic device over 2 decades ago. Preclinical data with the AngioJet confirmed that the heart block was the result of release of adenosine from hemolyzed blood. The effluent from the AngioJet was found to cause heart block and aminophylline prevented the block while atropine had minimal effect [4,5]. Historically, temporary pacemakers were placed prior to atherectomy in higher-risk situations (dominant right or left circumflex coronary arteries, etc) in order to treat this complication when it occurs. However, as atherectomy techniques advanced, some operators described attempts at avoiding routine temporary venous pacing by managing transient bradycardia with atropine, aminophylline, or vagolytic maneuvers [6]. However, none of these techniques have been systemically studied.

In this issue, Nakhle, et al. present a retrospective single-center analysis evaluating the safety and effectiveness of routine use of intravenous aminophylline (an adenosine receptor antagonist) during orbital and rotational atherectomy in an attempt to avoid significant bradycardia during atherectomy runs [7]. They evaluated 138 cases from 2018 to 2021 where intravenous aminophylline was prophylactically administered during either rotational or orbital atherectomy in order to avoid need for temporary pacing. They reported no adverse events related to aminophylline use. Still, 25 of 138 patients (18 %) required intravenous administration of atropine and 4.3 % required insertion of a temporary pacemaker. Notably, all those requiring a temporary pacemaker were undergoing atherectomy of a dominant right coronary artery. Also of note, while there were no reported deaths in this registry although their reported major adverse event rate was higher than has previously been reported [6].

The prevalence of calcified coronary lesions continues to rise and therefore atherectomy will continue to be a necessary tool in the Interventional Cardiologist's toolbox for treating significantly calcified lesions. The most recent Expert Consensus Statement on Management of Calcified Coronary Lesions Requiring Intervention released by the Society for Cardiovascular Angiography & Intervention (SCAI) recommends the use of atherectomy for uncrossable lesions and long, diffusely calcified lesions [2]. The current article certainly provides encouraging information about the safety of routine use of intravenous aminophylline during atherectomy in order to minimize significant bradycardia, though unfortunately breakthrough cases requiring atropine and/or temporary pacing still existed in their registry. Further research is required to continue to refine atherectomy techniques to improve outcomes and safety metrics.

## Funding

None.

## CRediT authorship contribution statement

**Robert F. Riley:** Resources, Visualization, Writing – original draft, Writing – review & editing. **Timothy D. Henry:** Project administration, Supervision, Validation, Visualization, Writing – original draft, Writing – review & editing.

## Declaration of competing interest

None.
